# Enhancing the wellbeing of refugees living with advanced life-limiting illness in high-income resettlement countries: A systematic review

**DOI:** 10.1177/02692163251338583

**Published:** 2025-06-14

**Authors:** Merrington H, Mahimbo A, DiGiacomo M, Roxas-Harris B, Agar MR, Nathan S, Hayen A, Heywood AE, Dawson A

**Affiliations:** 1 School of Public Health, The University of Technology Sydney, Sydney, NSW, Australia; 2 IMPACCT, The University of Technology Sydney, Sydney, NSW, Australia; 3 School of Population Health, UNSW Medicine & Health, The University of New South Wales, Sydney, NSW, Australia

**Keywords:** Refugees, palliative care, bereavement, systematic review

## Abstract

**Background:**

Refugees experience barriers to health care after resettlement and may have distinct palliative care needs. There is no systematic guidance to support person-centred palliative care services that are responsive to refugees’ needs and preferences.

**Aim:**

To synthesis evidence regarding factors enhancing the wellbeing of refugees with advanced life-limiting illness, and their families, to inform palliative care in high-income resettlement countries.

**Design:**

A systematic review of primary research studies. We applied a strength-based assets framework to the data extraction and synthesis and conducted a directed content analysis.

**Data sources:**

We searched nine electronic databases.

**Results:**

Ten of the 1006 studies identified were included in the review: two qualitative, one quantitative and seven case studies. We identified 17 assets that enhanced refugees’ wellbeing: resilience, religion, spirituality, sense of identity, belonging, community connections, health and death literacy, acculturation, family and social support, social capital, community structures, access to funeral information, access to services, palliative care service approaches, and workforce capacity. Resilience was linked to identity and belonging, connections within cultural and religious networks, social capital and creating meaningful funeral rituals in resettlement. Palliative care workforce capacity, death literacy, acculturation, refugees’ grief experiences and willingness to discuss and plan for death, influenced refugees’ attitudes to palliative care, communication with staff about treatment, prognosis and spiritual care, and care outcomes.

**Conclusions:**

Further research, co-designed with diverse refugee groups, is needed to inform palliative care service approaches, develop interventions to strengthen key assets and explore the nuanced role of social capital in end-of-life care.

**What is already known about the topic?**

- In high-income countries, refugees experience barriers to accessing health care that may delay palliative care seeking.
- Refugees’ cultural backgrounds and experiences of trauma, loss and grief during forced displacement shape health, wellbeing and expectations of care.
- Evidence is needed to inform palliative care services and approaches to supporting resettled refugees and their families.

**What this paper adds**

- This review demonstrates the dearth of research focused on resettled refugees living with advanced life-limiting illness and their families in high-income countries.
- The review highlighted the importance of assets such as resilience, sense of identity and belonging, community connections, social support and social capital, for enhancing the wellbeing of refugees and their families during end-of-life care and bereavement.
- Refugees’ cultural identity, death literacy and experiences of grief influence engagement with palliative care staff and decision-making about end-of-life care approaches.

**Implications for practice, theory or policy**

- Community networks play an important role in end-of-life care and bereavement support for refugees and their families.
- Participation of diverse groups of refugees in co-designed research is needed to build an evidence base to inform palliative care service approaches and develop community-based end-of-life care interventions that strengthen assets that enhance refugee wellbeing.
- Future studies should focus on refugees as a distinct group compared to migrants and the general population in high-income resettlement countries.

## Introduction

The global number of forcibly displaced people increased to 110 million in 2023, including more than 36 million refugees.^1^ One in 200 people in the world is a refugee,^2^ ‘someone who has been forced to flee their country because of war, violence or persecution, for reason of race, religion, political opinion or membership in a particular social group’.^3^ Most of the world’s refugees are hosted, at least temporarily, by Iran, Türkiye, Columbia, Germany and Uganda.^4^ The United States (US), Canada and Germany currently accept the highest numbers of refugees for resettlement through UNHCR-facilitated pathways.^4^

Little is known about the health and wellbeing of refugees after resettlement in high-income countries, including those diagnosed with a life-limiting illness, and the families and carers that will provide the bulk of care and bereavement support. Refugees may have distinct needs for palliative care. They may present to health services with more advanced illness because of lack of access to prevention and treatment, in refugee camps or countries where health services are fragile or non-existent.^5^ In high-income countries, refugees experience barriers to accessing health care due to lack of familiarity with services available, difficulties engaging with staff and concerns about trust.^6^ Many refugees have also experienced imprisonment, torture, violence, trauma, psychological distress and profound loss and grief. Experiences of forced displacement, and being separated from home and family, can have enduring effects on child and adult refugees’ mental health and wellbeing.^7^ The prevalence of anxiety, depression and post-traumatic stress disorder (diagnosed and self-reported) for child, adolescent and adult refugees residing in high-income countries, is reported to be substantially higher than in non-refugee populations globally or those living in war and conflict situations.^8^ During resettlement, refugees’ mental health challenges can be compounded by social determinants such as uncertainty about legal status, social isolation, stigma and discrimination, housing instability and financial stress.^9^ Research suggests that these types of experiences exacerbate refugee morbidity and mortality.^10^ Evidence is needed to inform approaches to palliative and end-of-life care that effectively respond to the mental health and other challenges refugees and their families confront.

Enhancing wellbeing is a fundamental goal of palliative and end-of-life care, and wellbeing has been described as the main indicator of quality of life in this context.^11^ While consensus is yet to be reached on conceptualisations of wellbeing and quality of life, they have evolved over time to become virtually synonymous.^12^ In high-income countries, palliative care service delivery focuses on person-centred care,^13^ advance care planning,^14^ end-of-life conversations with patients about their priorities^15,16^ and supporting people to die in their preferred place.^17,18^ Voluntary assisted dying, while not considered part of palliative care, is also expanding globally as an option for people with life-limiting illness.^19^ These approaches reflect the centrality of placing the patient and their family at the core of palliative and particularly end-of-life care. However, whether these approaches enhance the physical, mental and spiritual wellbeing of refugees and their families, or reflect their preferences and needs, is unknown.

Refugees who have resettled in high-income countries experience multiple challenges and are a potentially underserved group in terms of health care.^20,21^ Palliative care services need to respond appropriately to the complex needs of refugees with advanced life-limiting illness and their families, particularly the impact of refugees’ experiences of trauma, loss and bereavement. To achieve equity in end-of-life care outcomes, there is a need for evidence-based approaches to care that enhance the health and wellbeing of refugees and their families and reflect their priorities and care preferences. We sought to synthesise evidence of factors that enhance wellbeing for refugees with a life-limiting illness, and their families to inform palliative and end-of-life care in high-income resettlement countries.

## Methods

As part of the Health in a New Home study of refugee health,^22^ two authors (HM and AD) developed a systematic review protocol (Prospero ID: CRD42023493399) which all authors reviewed. A narrative synthesis design^23^ was adopted, to include diverse study types, including qualitative studies and more nuanced data related to refugees’ experiences of wellbeing, priorities and preferences. The review question was ‘what enhances wellbeing for refugees with an advanced life-limiting illness, their families and carers in high income resettlement countries?’ The review was reported according to the Preferred Reporting Items for Systematic reviews and Meta-Analyses (PRISMA).^24^

### Search strategy and study selection

The review included publications from 2003 to 29/02/2024, published in English or as an English translation. The year 2003 was chosen as the start date for two reasons. Firstly, this time frame was consistent with The Health in a New Home study, that focused on people of refugee background who resettled in Australia from 2003. In addition, we decided to limit the review to more recent studies, that would likely have been conducted in contexts more aligned with current approaches to health care and refugee resettlement. We anticipated that these studies would be more relevant for informing contemporary palliative care practice.

Inclusion and exclusion criteria are presented in Table 1. We included studies of refugees with advanced life-limiting illness, their families or carers living in high-income countries. We defined advanced life-limiting illness as a condition where a person is expected to die prematurely as a direct consequence of a specific illness or disease within the next 12 months.^25,26^ Conditions could include chronic cardiovascular, respiratory, kidney and liver disease, cancer, progressive neurological conditions and complications related to diabetes.

**Table 1.** Inclusion and exclusion criteria.

Study characteristic	Inclusion criteria	Exclusion criteria
Population	Adults, children and adolescent refugees with advanced life-limiting illness, and their families.	Asylum seekers Migrants and culturally and linguistically diverse groups unless results for refugees were disaggregated
Intervention or exposure	Attitude to death, dying and end-of-life End-of-life care experiences or priorities Palliative and end-of-life care (in hospital, aged care, hospice, community) Voluntary assisted dying Funerals and burials Bereavement	Bereavement after a death prior to resettlement
Study design	Observational or experimental studies Case studies and case reports Primary research reports	Protocols Reviews Books and book reviews Commentaries Studies of refugee mortality, morbidity and cause of death

We searched the following databases: MEDLINE, EMBASE, CINAHL, PsycInfo, Web of Science Core Collection, Scopus, Proquest Dissertations and Theses Global, and Dissertations and Overton. We developed the search strategy in consultation with university librarians with experience conducting systematic reviews, using keywords and index terms. The final search included only population and intervention-related concepts. Search terms included: ‘refugee*’, ‘forced migrant*’, ‘forcibly displaced’, ‘palliative care’, ‘end of life care’, ‘terminal care’, ‘hospice care’, ‘dying’, ‘attitude to death’, ‘advance care plan*’, ‘bereavement’, ‘bereavement counselling’, ‘funeral plan’, ‘funeral rites’, ‘compassionate communities’ and ‘voluntary assisted dying’. The full search strategy is described in Supplementary Table A.

We used Covidence to screen records, remove duplicates and identify included studies. Two reviewers independently completed all title, abstract and full-text screening (HM and AM) and a third researcher (AD) resolved any conflicts.

### Data extraction and synthesis

We applied Morgan’s assets framework^27^ to the data extraction and synthesis. Rather than focusing on deficits and health problems, Morgan’s framework places strengths, resources and ‘assets’ that protect and enhance wellbeing, at the centre of the interpretive lens. This framework is based on the theory of salutogenesis, that the experience of wellbeing is not predicated on the presence or absence of disease.^28^ Morgan’s strengths-based framework allows capture of a broad range of components of wellbeing, including the capacity within individuals, families and communities to enhance their own wellbeing at end-of-life. Assets operate at multiple levels: individual, family, community and population.^29^

*Individual* health assets are positive characteristics inherent to a person, including their beliefs and attitudes, self-esteem, identity, resilience, sense of purpose, sense of belonging and acculturation. These assets also include personal resources and skills that influence the way a person interacts with their environment, such as community connectedness, religion, spirituality, health literacy, visa type, access to education, employment, transport and housing. Familial assets relate to an individual’s place in the family, intergenerational solidarity, gender norms, parenting approaches and connections with their families’ support networks and affinity groups. *Community* assets build collective efficacy and communal flourishing and include social support, social capital and supportive community structures such as community organisations and places of worship. Finally, *population* assets are outside an individual’s control such as legal, economic and social policies and accessibility of health services.^27,29^

Using NVivo, we extracted data from the results sections of included studies related to factors influencing the wellbeing of refugees, their families and carers, including participant quotes and author interpretation of data. We conducted a directed content analysis^30^ of extracted data, using Morgan’s model^27^ to inform the coding framework.^29^ We initially categorised extracted data as assets (parent nodes) and according to their level of operation (individual, familial, community or population-level) in different study contexts. Data related to each asset, or asset clusters, were then coded inductively. A selection of included studies were independently coded by two researchers (HM and AD) to compare codes and refine the coding framework. We used an iterative approach to group codes and construct themes in the synthesis, within asset clusters and across asset levels.

### Quality appraisal

Two researchers (HM and AM) conducted the quality appraisal and a third researcher (AD) resolved conflicts. We used the AXIS Tool for cross-sectional studies^31^ and the CASP Qualitative Studies Checklist^32^ for qualitative and case studies. We used three additional items^33^ to appraise case studies (Table 2), instead of other tools designed for detailed medical case studies and reports (Table 2). We did not exclude any studies on the basis of quality.

**Table 2.** Additional items for quality appraisal of case studies and reports^33^.

Item
Was the reason for case selection stated?
Was adequate contextual description provided?
Was adequate case description provided?

## Results

**Figure 1.**
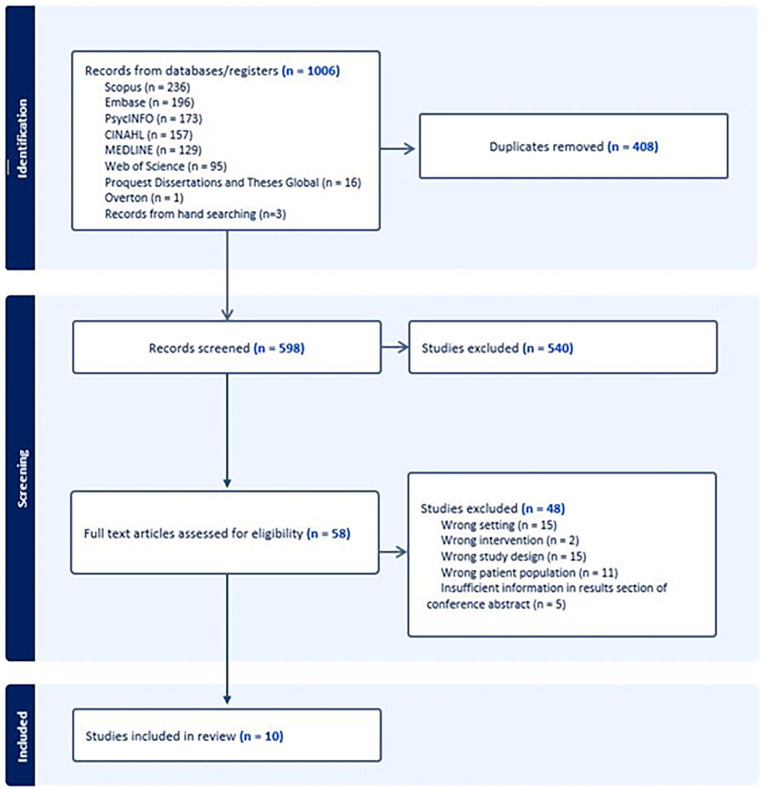
PRISMA flow chart.

Our search identified 1006 records and three additional studies were identified through hand searching reference lists of two relevant reviews.^34,35^ After duplicates were removed and title and abstract screening, full-text review of 58 records was completed (Figure 1). A total of 10 studies were included, published from 2011 to 2022 (Table 3).

**Table 3.** Included studies.

Study	Context	Study participants	Methodology	Aim	Main findings
**Abel** ^36^	UK	86-year-old woman, born Austria, 50+ years resettlement	Case study	To describe the family’s experience of creating a community support network.	The network enabled the woman with cancer to die at home and enhanced the well-being of family and friends, the woman and her husband.
**Bird** ^37^	Australia	Karen community leader; Information Day and funeral attendees.	Qualitative: Participant observation, interview	To explore death and dying in a Karen refugee community.	Death and dying was challenging for resettled refugees. Access to information about funeral regulations mobilised the community to take collective action to develop acceptable funeral rituals.
**Borneman** ^38^	USA	82-year-old man, born Eastern Europe, 50+ years resettlement	Case study	To describe an approach for providing spiritual care.	Conducting a spiritual history using the FICA tool created opportunities to provide wholistic care to a person dying from cancer.
**Hiruy and Mwanri** ^39^	Australia	Man in his 40 s, born Ethiopia, approx. 5 years resettlement	Case study	To inform culturally-appropriate palliative care.	It is preferable that family and community provide care at end of life, including religious and spiritual care to person dying from cancer.
**Hudson** ^40^	UK	Bosnian refugee community workers (*N* = 5); hospice staff.	Case study	To describe an approach to building community capacity to deliver end-of-life care.	Hospice staff trained five Bosnian community workers using the QELCA program. The approach needs to be adapted for refugees.
**Kokou-Kpolou et al.** ^41^	France and Belgium	Bereaved relatives born in Togo (*N* = 20), mean age 45.9 years, 62% male, mean duration of resettlement 16.75 years.	Cross-sectional survey	To describe and compare migrants’ and refugees’ complicated grief reactions in the context of migration trajectory.	Refugees experienced more complicated grief reactions than migrants (Mean ICG score: 40.2 vs 31.33, SD: 7.93 vs 5.88, *p* < 0.001). Duration of immigration was also significantly associated with complicated grief (adjusted *R*^2^ = .45, *F* = 31.09, *p* < .0001, η^2^ = .46)
**Kristiansen et al.** ^42^	Denmark	Bereaved spouse, woman aged late 50s, born Palestine, 20+ years resettlement	Case study	To explore how Islam and refugee experiences shape bereavement	Religious and refugee identity, connectedness to cultural and religious communities were sources of strength when her spouse died from cancer and during bereavement.
**Sneesby et al.** ^43^	Australia	Bereaved relatives (*N* = 15), 18–53 years, born South Sudan, duration of resettlement (0.5–6 years).	Qualitative: Focus groups	To inform palliative care service provision for Sudanese refugees.	Families’ cultural and religious beliefs influenced communication with health staff and preferences for end-of-life care. Talking about death and dying were often taboo and community members provided grief support for the family.
**Stahnke and Cooley** ^44^	US	96-year-old man, born Austria, 50+ years resettlement	Case study	To explore the use of narrative therapy techniques by a hospice social worker	Narrative therapy techniques assisted the patient (with advanced heart failure) to find spiritual meaning, process trauma and experience less fear of death, depression and regret.
**Swetz et al.** ^45^	US	41-year-old man, born Somalia, 13 years resettlement	Case study	To explore challenges of providing end-of-life care	The man with cancer and his wife were reluctant to engage with staff about their preferences and did not accept recommended care.

Refugee cases had advanced cancer (*n* = 5) and heart failure (*n* = 1) and were aged in their 40 s to 90s. Four cases were men and five had been resettled for more than 10 years.

### Study characteristics

Of the 10 included studies, two were qualitative,^37,43^ one was quantitative^42^ and seven were single case studies.^36,38–40,42,44,45^ Qualitative and quantitative studies were of good quality. However, case study quality varied: Good *n* = 4^38,40,42,44^; Fair *n* = 2^39,46^; Poor *n* = 1,^45^ due to insufficient case description, limited or unclear reporting of data collection and analysis, and lack of ethics approval (Supplementary Tables C and D). We included one conference abstract as a case study^40^ and obtained additional nformation from the author.^47^

Of the seven case studies, six were authored by health professionals, one of whom was also a case’s family member and one by a researcher from the same religious faith. Six case studies described a refugee participant case and one,^40^ a training program as the phenomenon of interest or ‘case’.

There were no experimental studies or evaluations of interventions. Three studies were conducted in Australia, three in the US and four in Europe. Refugee participants were born in Eastern Europe, Burma, Palestine, Ethiopia, Togo, South Sudan and Somalia.

### Synthesis of results

**Figure 2.**
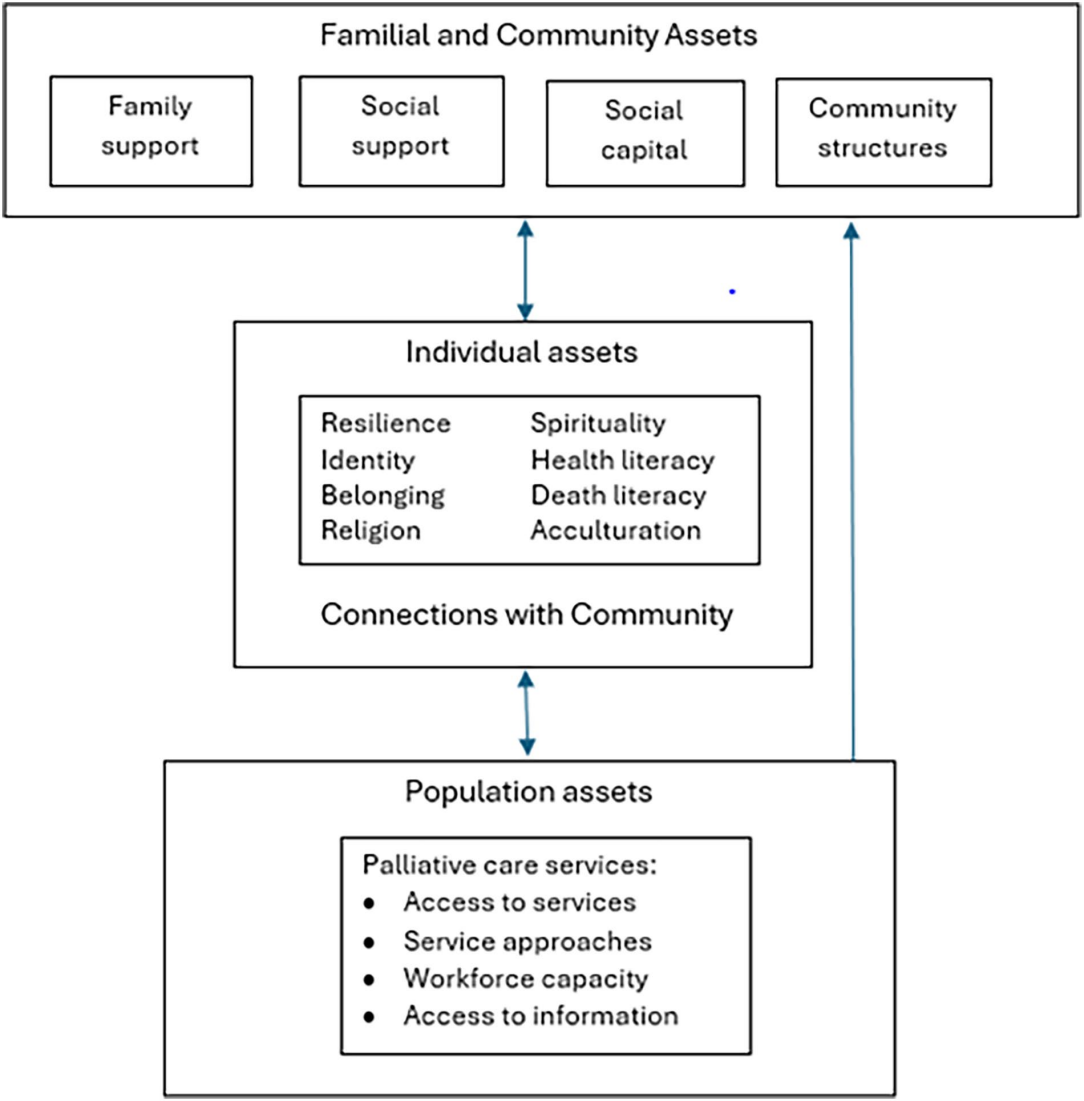
Asset clusters and linkages in end-of-life care and bereavement.

A total of 17 assets enhancing wellbeing for refugees living with advanced illness, their families and communities were identified in included studies (Figure 2). Nine of these were individual-level assets: sense of identity; sense of belonging; resilience; religion; spirituality; health and death literacy; acculturation and community connections. One asset, family support, was familial level and three were community-level, including social support, social capital and supportive community structures. The four population-level assets included access to services, palliative care service approaches, workforce capacity and access to death literacy information. Seven included studies found assets operating at all four levels (Table 4).

**Table 4.** Asset clusters and asset levels for included studies.

Study	Individual		Familial	Community	Population
Abel^36^	• Sense of belonging • Community connections		• Family support	• Social capital • Social support	• Service approaches
Bird^37^	• Resilience • Sense of identity • Sense of belonging • Community connections	• Religion • Death literacy • Acculturation	• Family support	• Social support • Social capital • Community structures	• Access to death literacy information
Borneman^38^	Spirituality				• Service approaches • Workforce capacity
Hiruy and Mwanri^39^	• Resilience • Sense of identity • Community connections • Religion		• Family support:	• Social support • Social capital	• Service approaches
Hudson^40^				• Social capital	• Service approaches • Workforce capacity
Kokou-Kpolou et al.^41^	• Resilience		• Family support	• Social support	• Access to service type
Kristiansen et al.^42^	• Resilience • Sense of identity • Sense of belonging	• Community connections • Religion	• Family support	• Social support • Social capital • Community structures	• Service approaches
Sneesby et al.^43^	• Resilience • Sense of identity • Community connections • Religion	• Spirituality • Health literacy • Death literacy • Acculturation	• Family support	• Social support • Social capital	• Service approaches • Access to service type
Stahnke and Cooley^44^	• Resilience • Spirituality • Death literacy		• Family support	• Social support	• Service approaches • Building workforce capacity • Access to service type
Swetz et al.^45^	• Health literacy		• Family support		• Service approaches

Overall, included studies focused on the following contexts: end-of-life care for refugees with advanced illness and their families (*n* = 8)^36,38–40,42–45^; bereavement (*n* = 5)^37,39,41–43^ and both contexts.^39,42,43^ The same assets were identified across both contexts with one exception: palliative care service approaches were not included in bereavement studies.

### End-of-life care

#### Individual assets: Resilience, religion, sense of identity and community connections

The asset of resilience was a dominant theme in the synthesis for enhancing wellbeing for the dying person and their family in the context of end-of-life care. Resilience was often linked with a cluster of individual assets, including religion, spirituality, community connections, sense of identity and belonging.

In three studies, religion was linked with resilience, enhancing wellbeing by offering reassurance and comfort for those approaching their own death or a family member’s:

*When they told [my husband] you have cancer. It was a shock for me. . . . Then when the doctor left, I cried, [and] I told my husband ‘Please don’t leave this life before me, pray for me that Allah will be with me. . . don’t go without sending me to Allah’.*^42^ (p. 232)

Prayer and religious faith were sources of strength: *‘If you are sick and can’t get better, pray (Joseph)’*^43^ (p. 2700). However, some participants with advanced illness expressed ambivalence about their religion or rejected it after their experiences as refugees, when they ‘*struggled to make sense of a world where a God could allow for the horrors [they] had witnessed*’.^44^ (p. 193) These participants drew on other sources of spirituality and meaning for resilience, such as legacy and family:

Frank felt a sense of pride for the life he had lived. . . Due to the emphasis on his legacy and passing on of wisdom to a younger generation, Frank felt he was leaving himself behind in his family, friends, and contributions.^38,44^ (p. 195)

Resilience was also linked to the assets of sense of identity and community connections. Cultural or religious identity and close connections within affinity networks could shape customs and priorities related to end-of-life care. Subscribing to these practices and fulfilling cultural obligations could enhance wellbeing for the dying person and their family. For example, having friends and community members visit the dying person just prior to death, was a priority for some Ethiopian, Sudanese and Palestinian refugees^39,42,43^: *‘Various spiritual and religious leaders spend time with the person. . . this is also considered as the right time to reconcile with people and God’*.^39^ (p. 4)

In two studies, sense of belonging was linked to a person’s refugee identity.^36,42^ The loving presence of family and social support at the end-of-life affirmed connection and belonging, a powerful antidote for past traumatic experiences of alienation and loss:

*My mother was more peaceful and content during this period than I had ever seen her. It was as if the burden she carried, of searching for a sense of belonging had lifted. She had found it, among the love and care of family and friends*.^36^ (p. 2)

#### Resilience, family and social support

The role of family support and social support assets in enhancing resilience and wellbeing was another major theme in the synthesis. Families and, in many cases, friends and community members offered support by providing practical and emotional care to the person who was dying or family members: ‘*His family was always beside him, and it was not easy for them to let him be cared for by others at the palliative care ward*’.^39^ (p. 4) Family and community support could allow a person to die at home: ‘*We asked her, after she had been home for a couple of weeks, if she still wanted to die in the hospice. She said that she actually felt really comfortable at home and if it was ok with everyone [in the care network], she would like to die there, which she did peacefully*’.^36^(p. 2)

Cultural and religious community members sometimes provided spiritual care for someone dying at home or in hospital, including those with diminished family networks: ‘*If they do not have an adult son, the community leader takes on that responsibility*’.^43^ (p. 2698) Communities also assisted in fulfilling cultural or religious obligations and customs related to end-of-life care:

*When Dagne was hospitalised, many people travelled to see him from across Australia – to pay their last respect to make things right before ‘God takes him away’.*^39^ (p. 4)

In this way, assets such as resilience, identity and community connections were linked with family and social support. However, social norms and religious customs could also diminish resilience, particularly for carers. In one case study of a Palestinian refugee, fulfilling religious obligations to her family and community caused her to ‘*neglect herself*’^42^ (p. 235):

*Although support was increased from members of the religious community during her husband’s illness, she struggled to uphold her identity as a wife and a mother throughout the process . . . her distress revolved around the management of conﬂicting responsibilities toward her husband and her children.*^42^ (p. 233)

#### Social capital

Social capital was a community asset that could enhance wellbeing through building capacity for community-based end-of-life care support networks for families. In one case study, establishment of a 24-hour ‘circle of care’ network, comprised of family and friends, made dying at home an option for the author’s mother^36^ and allowed family and community members providing care to support each other. This network was later deployed to care for her spouse when he was dying.

However, building social capital for end-of-life care was dependent on access to supportive population assets in the form of adequate training for volunteers (workforce capacity) and the use of approaches acceptable to the community (service approaches). In one case study,^40^ an attempt by hospice nurses to deliver the ‘Quality End-of-Life Care for All’ training program^48^ to volunteer community workers from the Bosnian refugee community in the United Kingdom, was hampered by lack of trust, differing expectations and goals, and lack of acceptability of some approaches to end-of-life care included in the training.^47^

#### Health and death literacy and palliative care service approaches

Health and death literacy were important individual assets for wellbeing, for people who were dying and their families. For example, health literacy influenced families’ expectations of health care: ‘*Participants had the perception that in Australia, medicine is sophisticated and treatment can cure almost anything. They expressed disappointment in their experiences of the Australian healthcare system*’.^43^ (p. 2699) In two studies, death literacy, defined as awareness or understanding of palliative care approaches,^49^ influenced attitudes and decision-making about end-of-life care for Sudanese and Somali-background families^43,45^: ‘*When you turn off the machine that is keeping someone alive, that is killing*’.^43^ (p. 2699) Some Sudanese refugees’ explanatory models of illness and death influenced attitudes to the timing of palliative care treatment-seeking and the use of traditional and western medicines: ‘*In Sudan, the elders are hospitalised only at the very end of life for terminal care*’.^43^ (p. 2699) However, in this study, attitudes and knowledge varied across generations, according to age, education and time spent in Sudan and Australia, suggesting the asset of acculturation also influenced health and death literacy:

*The younger participants (under 30 years) . . . wanted to be fully informed of all healthcare issues about themselves and their families. They did not want the involvement of the community in their healthcare decision-making*.^43^ (p. 2698)

Willingness to discuss and plan for death and dying was another aspect of death literacy that influenced wellbeing. In two studies, discussions about dying enhanced wellbeing for the dying person^36,44^:

*I provided him education on the dying process . . . so that he could rest assured that his death would be peaceful with hospice care . . . he expressed decreased fear around death overtime and even “acceptance of what’s coming*.”^44^ (p. 195)

In contrast, cultural ‘taboos’ and active avoidance of discussions about death and dying could reinforce low death literacy. For some Sudanese refugees, taboos about discussing death and dying were a form family support and protection: ‘*Death and dying is not discussed openly. . . This will make them sad and they will maybe die. . . . (Betty)*’.^43^ (p. 2698)

Avoiding discussions about dying could also affect outcomes of care. In one case study, a Somali man presented at a hospital emergency department with severe back pain related to advanced cancer^45^ and died a month later:

*He did not appear to be open to discussing the severity of his medical situation, nor did he wish to discuss end-of-life preferences. The social worker reported that he responded to her questioning, “I don’t know, I don’t know, talk to my wife”. [A]fter two weeks in our facility, the man did agree to go to [a] rehabilitation facility. A follow-up appointment with oncology was scheduled to discuss advanced line chemotherapy. The patient never made it to that appointment. He died three weeks after discharge from our hospital*.^45^ (p. 44)

Health and death literacy were also linked with population assets such as workforce capacity. The way staff approached engaging with families with low health and death literacy had the potential to have a major impact on the wellbeing of the patient and their family. Treatment goals and the purpose of palliative care interventions needed to be explained to family members to put them at ease.^43^ In cases where direct discussions about death with the dying person were avoided, it was necessary for staff to establish who was the designated intermediary (e.g. their spouse or family friend) for discussing end-of-life care.^42,43,45^ Staff awareness of some families’ deference to medical authority^43,45^ and how refugees’ experiences of trauma, uncertainty and forced displacement influenced their reactions to death and dying^44^ was also beneficial. Staff training was needed to build workforce capacity for communicating in these ways, to support effective service delivery.

Approaches to spiritual care in hospital was another population asset linked to resilience in three studies.^38,39,44^ It was recommended that family and community members, who were important sources of spiritual care, be documented in hospital end-of-life care plans. Developing strategies for managing large numbers of family and community members on palliative care wards as part of spiritual care, was also recommended.^39^ Access to hospice care was a desirable option for some Sudanese families:

*There is a belief that when a person dies their spirit remains in that place. Some people do not want the spirit to linger around the home as this may cause fear. They had never heard of palliative care and welcomed the idea of a hospice*.^43^ (p. 2699)

Spiritual assessment by health professionals, using validated tools could assist in identifying patients’ spiritual needs and sources of resilience,^38^ particularly for those without religious affiliations.^44^ The value of strength-based approaches such as narrative therapy techniques was highlighted in one case study for building resilience and working with refugees’ trauma and fear of death.^44^

### Bereavement

#### Resilience, sense of identity and social support

The cultural importance of mourning rituals, carried out immediately following a person’s death, and funerals for resilience, was a strong theme in the synthesis.^37,39,41–43^ Upholding mourning customs was part of honouring the person who died and coping with grief. One study reported that Togan refugees experienced more complicated grief reactions compared to Togan migrants and suggested this was linked to refugees’ capacity to participate in funeral rituals.^41^ In this study, returning a relative’s remains to their birth country could be a barrier to funeral participation for refugees who could not afford travel, or enter the country safely.

In other studies, resilience was linked to sense of identity, belonging, connections to community, religion and spirituality,^37,39,41–43^ as was the case in the end-of-life care context. Immediately after a death, family and cultural or religious community members played specific roles: ‘*the body is kept at home, waiting for relatives to arrive, which can take up to three days. There is always somebody talking, singing or praying with the body, day and night*’.^37^ (p. 450)

Family and social support protected the wellbeing of bereaved family members. For some Ethiopian and Sudanese refugees, expressions of grief were highly demonstrative, taking the form of screaming, loud wailing and crying, at the family home or in the hospital.^39,43^ Some Sudanese refugees’ reactions involved physically hurting themselves: ‘*One participant related the story of her cousin, who on being told of her father’s death, repeatedly banged her head on the wall, then threw herself on the ground hitting her head and sustaining a fatal head injury*’.^43^ (p. 2698) Notifying older family members of ‘bad news’ was postponed until community, spiritual or religious leaders were present, to protect family members from self-harm or suicide, particularly after the death of a child:

*Gathered community members protect the family for three days. . . Initially, furniture is removed from the family home and mattresses are arranged on the floor. Grieving families are never left alone.*^43^ (p. 2698)

While participation in mourning practices was a form of social support, the presence of mourners in the family home for extended periods could also create significant burdens for families, in the form of *‘significant health and economic impacts’*.^39^ (p. 5)

#### Resilience and social capital

Funerals, as demonstrations of ethnic or religious identity, affirmed a sense of belonging and resilience for coping with grief. Gathering with the Palestinian Muslim community was described in one case study as creating solidarity ‘*strengthened by life in exile*’.^42^ (p. 229) In Bird’s ethnographic study of a Karen funeral, the event offered refugees continuity and connection with the past and the homeland, providing temporary ‘cultural familiarity’^37^ and comfort for the bereaved. Funerals were ‘transnational practices’, created through blending traditional customs with new practices, within the realities and constraints of the resettlement context. In this way, transnational practices built social capital, described as a ‘*tactical. . . solution to the problem of feeling out-of-place*’ (p. 455). Transnational practices could also be linked to acculturation, supporting possibilities for adjusting traditional customs and rituals.

Only two studies focused on bereavement beyond the period immediately following a death. Both studies focused on resilience, social support and social capital, emphasising the role of ethnic and religious community members in providing comfort for grieving families.^42,43^ For a bereaved Muslim refugee in Denmark, religion was central to creating meaning in her life.^42^ Praying with other Palestinian refugees at the Mosque after the death of her husband was a source of comfort and an asset for avoiding the ‘*isolation of widowhood*’.^42^ (p. 2016) In addition, as a community space, the Mosque supported maintenance of connections with her religious, cultural and refugee identity, as she ‘*reconstructed. . . everyday life following the death of her husband*’.^42^ (p. 235)

#### Social capital, death literacy and self-determination

Bird’s study described examples of how social capital assets could be mobilised for self-determination and community action to enhance wellbeing.^37^ Strong community leadership and enhancing death literacy drove action to prepare for funerals and burials within the Australian Karen community. Tailored information about funeral and burial regulations was provided by a funeral director at a Community Information Day to enhance death literacy. The community then acquired burial plots for their community and develop a new funeral format, that was later shared with other ethnic refugee groups from Burma.

## Discussion

Our review highlighted a dearth of research on end-of-life care for refugees living with advanced illness and their families in high-income countries. However, despite the small number of included studies, we identified 17 assets that enhanced refugees’ wellbeing, operating at individual, familial, community and population levels. These findings can inform future research aimed at developing interventions that strengthen these assets and enhance wellbeing for refugees living with advanced illness and their families.

Assets were context-dependent and interconnected rather than operating as discrete units. Linkages between assets created clusters within and across asset levels, that broadened their scope of influence by working together. Some assets could enhance wellbeing in some circumstances and diminish it in others. Future studies also need to examine contextual factors that constrain or enable assets-based community capacity building and empowerment.^50^

### Resilience, identity and social capital

Many identified assets fostered resilience. While the link between social support, psychological wellbeing and resilience is well-established,^51^ our review highlighted the particular importance of assets like identity and belonging for resilience, and the role of strong connections within cultural and religious community networks, in end-of-life care and bereavement for refugees in resettlement contexts. This is consistent with previous research on assets-based community development approaches to building refugees’ resilience by reducing social isolation.^52^

Religious and cultural identity could be a source of strength for people with advanced illness and their families and mobilise communities to provide support with end-of-life care. However, cultural and religious obligations could also diminish resilience by overwhelming some family members with caring responsibilities, particularly women.^42^ Bonding social capital, or strong ties within groups with similar characteristics, may offer support but, in some circumstances, also negatives in the form of social influence and control that can disadvantage or marginalise some groups.^53^ Some studies suggest that bonding social capital is most beneficial in the first years of resettlement^54^ and that ‘bridging’ social capital, based on connections between those with different identities but similar positions of power,^55^ may also be important for enhancing refugees’ mental health outcomes and resilience.^56^ Broadening refugees’ community ties to encompass connections both within and outside of cultural or religious networks, may offer access to alternative forms of support and resilience.^57^ Given the heterogeneity of refugee communities, understanding the nature of a family’s existing community connections would be valuable for planning how best to support them. Further research is also needed, that explores the role of social support and different forms of social capital in end-of-life care and bereavement for diverse groups of refugees.

### Social capital and community empowerment

The significance of mourning rituals and funerals for the wellbeing of refugees and their families was also highlighted in the review. Social capital^58^ could drive a collective process of adjustment to death in the resettlement context, when there was strong leadership and community mobilisation to ‘take ownership of death and dying’.^37^ Social capital could also facilitate acculturation, when culturally-meaningful mourning practices were created in a new context. In this way, acculturation could be a gentler and more active, shared process of finding ways to cope with loss and grief.

The role of community in end-of-life care was also an asset for refugees and their families. The value of death literacy as an asset for empowering refugee communities to build their capacity to deliver community-based end-of-life care, was also a theme of the review. This finding builds on existing research, demonstrating how community involvement can have a positive impact on end-of-life care by offering practical support, building community capacity, improving outcomes for carers, influencing place of death and the involvement of palliative care services.^59,60^ The Compassionate Communities model^61^ is an example of this approach with potential utility in communities where cultural differences in approaches to end-of-life care exist.^62^ However, our review also highlighted the need to develop acceptable training approaches, tailored to the needs of refugees.^47^ Overall, use of this approach with refugee communities warrants further investigation. In addition, connecting existing population assets through the sharing of resources between organisations, or building the capacity of community leaders to drive change, may be needed to enable assets like social capital to thrive.^63^

### Palliative care approaches and death literacy

Our review found that in high-income countries, palliative care service approaches, as population-level assets, played a vital role in supporting the wellbeing of the person with advanced illness and their family. Our findings were consistent with previous palliative care research involving migrants,^64^ reinforcing how lack of awareness of palliative care,^65,66^ low health and death literacy^67^ and cultural differences between patients, their families and health professionals^34,35,68,69^ influenced wellbeing. In addition, our review also suggested that some approaches to palliative care in high-income resettlement countries may not be acceptable to some refugees, particularly advanced care planning,^14^ a central tenet of person-centred palliative care.^13^ A patient or family’s reluctance to discuss prognosis and end-of-life care presents significant challenges for palliative care staff.^34^ While avoidance of death preparation is not unique to refugees,^70,71^ a history of trauma can present an additional barrier to acceptance of death, as well as increasing distress, exacerbating pain symptoms and mistrust of service providers.^72^ Studies on culturally-sensitive health service provision typically recommend that staff enhance their capacity to communicate better with families and bridge the gap of cultural difference and low health literacy.^6^ Based on our review, understanding how beliefs relate to assets and the functions they serve for families may assist palliative care staff in engaging and collaborating with families in a respectful and productive way. However, the voices of people of refugee backgrounds have been absent from palliative and end-of-life care research and future studies conducted in partnership with refugees are needed, to co-design acceptable and effective approaches to end-of-life care and communication with families.^13^

It is rare for researchers to focus on refugees as a distinct group with unique needs for end-of-life care. Our review confirmed some refugees’ lack trust in health service staff after past experiences of trauma and forced displacement.^34,35^ However, the impact of refugees’ repeated experiences of loss and grief on their reactions to a diagnosis or death in the resettlement country, was also highlighted, suggesting this is another area where some refugees differ from other population groups. Incorporating approaches for supporting loss and grief, including recognising the impact of cultural bereavement^73^ and ‘compounded traumas’^74^ may be beneficial for enhancing palliative care. Our review also suggested community can play a key role for many people from refugee background in responding to grief. Social support and cultural connectedness are important assets for bereaved families, when memories of the death of other family members, diminished family networks and the reality that they may never return to their home country in safety, are revived following the death of a partner, child or other family member.

### Strengths and limitations

The review was the first that has focused specifically on assets enhancing wellbeing for refugees living with advanced life-limiting illness and their families in high-income resettlement countries. Our use of an assets framework for conceptualising wellbeing broadened the review’s focus to incorporate strengths that exist within individuals, families and communities. As a result, our findings differ from two previous reviews involving refugees that largely focused on culturally-sensitive palliative care,^34,35^ by shedding light on refugees’ end-of-life experiences in community contexts. Our review also identified assets that were existing strengths that could be enhanced when combined with supportive population assets. While it is possible we made errors in our interpretation of assets in the data extraction, every effort was made to refine the specificity of the coding framework and maintain consistency between those coding and interpreting the data.

The lack of studies in this area was the primary limitation of our review. Researchers’ inconsistent use of terminology such as refugees, migrants and immigrants, hampered our search strategy and capacity to locate relevant studies related to refugees. No experimental or intervention studies were identified and more than half of included studies were exploratory case studies authored by health professionals and researchers, and were of varying quality. Despite this, the inclusion of case studies was a strength for exploring interactions between groups of people, and between assets and contextual factors influencing wellbeing. Exploring how assets are influenced by age, gender and duration of resettlement is also important in future studies, in recognition of the diversity of refugee communities.

The lack of research involving refugees as active participants in design, data collection and analysis was a major constraint on our capacity to understand refugees’ lived experiences and needs for end-of-life care and to what extent these differed from migrants or the general population. This can only be addressed through future mixed methods co-designed research involving refugees living with advanced illness and their families.

## Conclusions

The review found the development of an evidence base for end-of-life care for refugees is in its infancy. Our findings explored a range of assets enhancing wellbeing for refugees living with advanced illness and their families. However, further research is needed to understand contextual factors influencing assets and the needs of diverse groups of refugees living in high-income resettlement countries. As well as enhancing refugees’ interactions with palliative care services, the review’s findings highlight the need for approaches to support and build the capacity of refugee family and community networks to provide care during end-of-life and bereavement. Further research co-designed with refugees, is needed, to inform palliative care service approaches, develop effective community-based interventions and explore in a more nuanced manner the role of assets like social capital in promoting and protecting wellbeing.

## Supplemental Material

sj-docx-1-pmj-10.1177_02692163251338583 – Supplemental material for Enhancing the wellbeing of refugees living with advanced life-limiting illness in high-income resettlement countries: A systematic review Supplemental material, sj-docx-1-pmj-10.1177_02692163251338583 for Enhancing the wellbeing of refugees living with advanced life-limiting illness in high-income resettlement countries: A systematic review by Merrington H, Mahimbo A, DiGiacomo M, Roxas-Harris B, Agar MR, Nathan S, Hayen A, Heywood AE and Dawson A in Palliative Medicine

sj-docx-2-pmj-10.1177_02692163251338583 – Supplemental material for Enhancing the wellbeing of refugees living with advanced life-limiting illness in high-income resettlement countries: A systematic review Supplemental material, sj-docx-2-pmj-10.1177_02692163251338583 for Enhancing the wellbeing of refugees living with advanced life-limiting illness in high-income resettlement countries: A systematic review by Merrington H, Mahimbo A, DiGiacomo M, Roxas-Harris B, Agar MR, Nathan S, Hayen A, Heywood AE and Dawson A in Palliative Medicine

sj-docx-3-pmj-10.1177_02692163251338583 – Supplemental material for Enhancing the wellbeing of refugees living with advanced life-limiting illness in high-income resettlement countries: A systematic review Supplemental material, sj-docx-3-pmj-10.1177_02692163251338583 for Enhancing the wellbeing of refugees living with advanced life-limiting illness in high-income resettlement countries: A systematic review by Merrington H, Mahimbo A, DiGiacomo M, Roxas-Harris B, Agar MR, Nathan S, Hayen A, Heywood AE and Dawson A in Palliative Medicine

sj-docx-4-pmj-10.1177_02692163251338583 – Supplemental material for Enhancing the wellbeing of refugees living with advanced life-limiting illness in high-income resettlement countries: A systematic review Supplemental material, sj-docx-4-pmj-10.1177_02692163251338583 for Enhancing the wellbeing of refugees living with advanced life-limiting illness in high-income resettlement countries: A systematic review by Merrington H, Mahimbo A, DiGiacomo M, Roxas-Harris B, Agar MR, Nathan S, Hayen A, Heywood AE and Dawson A in Palliative Medicine
