# Supplementary material for: Enhancing the wellbeing of refugees living with advanced life-limiting illness in high-income resettlement countries: A systematic review
Source: Palliat Med. 2025 Jun 14;39(7):750–64. doi: 10.1177/02692163251338583 (PMC12227814; doi:10.1177/02692163251338583)
Supplement: sj-docx-1-pmj-10.1177_02692163251338583 – Supplemental material for Enhancing the wellbeing of refugees living with advanced life-limiting illness in high-income resettlement countries: A systematic review [file sj-docx-1-pmj-10.1177_02692163251338583.docx]

Supplementary File A: Search strategy

Medline

exp Refugees/

or

("refugee*" or "forced migrant*" or "forcibly displaced").mp. [mp=title, book title, abstract, original title, name of substance word, subject heading word, floating sub-heading word, keyword heading word, organism supplementary concept word, protocol supplementary concept word, rare disease supplementary concept word, unique identifier, synonyms, population supplementary concept word, anatomy supplementary concept word]

and

exp Palliative Care/

or

exp Terminal Care/

or

exp Hospice Care/

or

exp Attitude to Death/

or

exp Advance Care Planning/

or

exp Bereavement/

or

exp Suicide, Assisted/ or exp Euthanasia, Active, Voluntary/

or

("palliative care" or "terminal care" or "end of life care" or "hospice care" or "hospice*" or "dying" or "attitude to death" or "advanced care plan*" or "funeral plan*" or "funeral rites" or "bereavement" or "compassionate communities" or "voluntary assisted dying").mp. [mp=title, book title, abstract, original title, name of substance word, subject heading word, floating sub-heading word, keyword heading word, organism supplementary concept word, protocol supplementary concept word, rare disease supplementary concept word, unique identifier, synonyms, population supplementary concept word, anatomy supplementary concept word]

Embase

exp Refugees/

or

exp forced migrant/

or

("refugee*" or "forced migrant*" or "forcibly displaced").mp. [mp=title, abstract, heading word, drug trade name, original title, device manufacturer, drug manufacturer, device trade name, keyword heading word, floating subheading word, candidate term word]

and

exp palliative therapy/

or

exp terminal care/

or

exp dying/

or

exp bereavement/ or exp bereavement support/

or

exp hospice care/ or exp attitude to death/ or exp hospice/

or

exp posthumous care/

or

exp assisted suicide/ or exp voluntary euthanasia/ or exp euthanasia/

or

("palliative care" or "palliative therapy" or "end of life care" or "terminal care" or "hospice care" or "hospice" or "attitude to death" or "dying" or "bereavement" or "bereavement support" or "compassionate communities" or "advanced care plan*" or "funeral plan*" or "funeral rites" or "euthanasia" or "voluntary assisted dying" or "posthumous care").mp. [mp=title, abstract, heading word, drug trade name, original title, device manufacturer, drug manufacturer, device trade name, keyword heading word, floating subheading word, candidate term word]

CINAHL

(MH "Refugees+")

or

TI ("refugee*" OR "forced migrant*" OR "forcibly displaced")

or

AB (TI "refugee*" OR "forced migrant*" OR "forcibly displaced")

and

(MM "Palliative Care") OR (MM "Palliative Care Nursing") OR (MM "Palliative Care Nurses") OR (MM "Hospice and Palliative Nurses Association")

or

(MH "Terminal Care+") OR (MH "Terminally Ill Patients+")

or

(MM "Hospice Care") OR (MM "Hospices") OR (MM "Hospice Nursing") OR (MM "Hospice Patients")

or

(MM "Dying Process (Saba CCC)") OR (MM "Dying Care (Iowa NIC)") OR (MM "Dignified Dying (Iowa NOC)") OR (MH "Attitude to Death+")

or

(MM "Advance Care Planning") OR (MH "Advance Directives+")

or

(MH "Bereavement+") OR (MM "Bereavement Support (Saba CCC)")

or

(MM "Suicide, Assisted")

or

Funeral rite*: (MM "Burial Practices") OR (MM "Funeral Arrangements (Saba CCC)")

or

TI ("palliative care" OR "end of life care" OR "terminal care" OR "hospice care" OR "hospice*"OR "dying" OR "attitude to death" OR "advanced care plan*" OR "funeral plan*" OR "funeral rite*" OR "bereavement" OR "compassionate communities" OR "voluntary assisted dying")

or

AB ("palliative care" OR "end of life care" OR "terminal care" OR "hospice care" OR "hospice*"OR "dying" OR "attitude to death" OR "advanced care plan*" OR "funeral plan*" OR "funeral rite*" OR "bereavement" OR "compassionate communities" OR "voluntary assisted dying")

PsycInfo

MM “refugees”

or

"refugee*" OR "forced migrant*" OR "forcibly displaced"

and

DE "Palliative Care" OR DE "Assisted Suicide" OR DE "Euthanasia" OR DE "Hospice"

or

MM "Hospice" OR MM "Terminal Cancer"

or

MM "Advance Directives"

or

DE "Death and Dying" OR DE "parental death" OR DE "partner death" OR DE "terminally ill patients"

or

DE "Bereavement" OR DE "Grief"

or

MM "Death Attitudes"

or

TI ( "palliative care" OR "dying" OR "attitude to death" OR "terminal care" OR "hospice care" OR "advanced care plan*" OR "compassionate communities" OR "bereavement" ) OR AB ( "palliative care" OR "dying" OR "attitude to death" OR "terminal care" OR "hospice care" OR "advanced care plan*" OR "compassionate communities" OR "bereavement" )

Web of Science

**("refugee*" OR "forced migrant*" OR "forcibly displaced") AND ("palliative care" OR "end of life care" OR "terminal care" OR "hospice care" OR "dying" OR "attitude to death" OR "advanced care plan*" OR "compassionate communities" OR "bereavement" OR "voluntary assisted dying" OR "funeral plan*" OR "funeral rite*")**

**Scopus**

"refugee*" OR "forced migrant*" OR "forcibly displaced"

and

"palliative care" OR "end of life care" OR "terminal care" OR "hospice care" OR "hospice" OR "dying" OR "attitude to death" OR "advanced care plan*" OR "compassionate communities" OR "bereavement" OR "funeral plan*" OR "funeral rite*" OR "voluntary assisted dying"

**Proquest Dissertations & Theses Global**

abstract("refugee*" OR ("forced migrants") OR "forcibly displaced") AND abstract("palliative care" OR "end of life" OR "hospice care" OR "terminal care" OR "dying" OR "advanced care plan*" OR "compassionate communities" OR "attitude to death" OR "bereavement" OR “voluntary assisted dying”OR “funeral plan*”OR “funeral rite*”)

Overton

refugee* Topics: palliative care; end of life care. Post 1/1/2003 Sources: all except cities, states, hansards

or

Then: title: refugee* with selected topics, time frame and sources
