# Supplementary material for: Enhancing the wellbeing of refugees living with advanced life-limiting illness in high-income resettlement countries: A systematic review
Source: Palliat Med. 2025 Jun 14;39(7):750–64. doi: 10.1177/02692163251338583 (PMC12227814; doi:10.1177/02692163251338583)
Supplement: sj-docx-2-pmj-10.1177_02692163251338583 – Supplemental material for Enhancing the wellbeing of refugees living with advanced life-limiting illness in high-income resettlement countries: A systematic review [file sj-docx-2-pmj-10.1177_02692163251338583.docx]

Supplementary Table B: Codebook for assets in included studies

| Name | Description |
| --- | --- |
| Individual-level assets | |
| Community connections | Access to community connections based on cultural, religious affinity or other networks, that influences wellbeing  Factors influencing access to these networks  How they influenced wellbeing, either directly stated or implied |
| Religion | Religious beliefs or practices that influence well-being  Examples of circumstances when religious beliefs enhanced wellbeing  How they influenced wellbeing, either directly stated or implied |
| Spirituality | Beliefs related to spirituality (not connected to a specific religion), philosophical understandings or sources of meaning related to end of life, other non-religious sources of meaning and purpose at end of life.  How they influenced wellbeing, either directly stated or implied |
| Resilience | Beliefs, practices and contextual factors influencing coping, overcoming challenges or reacting to circumstances or events related to end-of-life care, bereavement, everyday life; sources of strength  How they influenced wellbeing, either directly stated or implied |
| Sense of belonging | References to sense of belonging, the desire to connect with others to feel accepted or included in a social group or community  How they influenced wellbeing, either directly stated or implied |
| Sense of identity | References to sense of identity, identity related to refugee experience, culture , ethnicity, religious faith, gender, age, occupation or any other personal characteristic  How they influenced wellbeing, either directly stated or implied |
| Health literacy | Knowledge of western medical model and related interventions, health services available, capacity to negotiate the system, capacity to communicate with health professionals via mechanisms offered within the system  Influence of individual explanatory models of health and illness  How they influenced wellbeing, either directly stated or implied |
| Death literacy | As for health literacy but specifically related to palliative care approaches and services, knowledge about end of life issues, care options, understanding of death and dying  Own explanatory models of death and dying  Willingness to talk about death and dying-reluctance, resistance or taboos about this; willingness to prepare for death eg. Advanced care planning  How they influenced wellbeing, either directly stated or implied |
| Acculturation | Examples of adjustment to a new context, bidirectional factors influencing blending of traditional and new ways of operating in resettlement, adjustments to life in resettlement that occur as a result of exposure to new service systems etc, adjustments in beliefs and practices, changes in values or identities  Contextual factors influencing acculturation-forced by context versus self determination or empowerment to create or accept new approaches  How they influenced wellbeing, either directly stated or implied |
| Familial-level assets | |
| Family support | Family support received or provided  Cultural norms, customs or obligations for families and who is responsible for them, whether some groups are burdened more than others, under what circumstances customs aren’t followed-and impact of this on access to family support  Factors influencing access to family support, tolerance of intergenerational differences in approaches  How these aspects enhance well-being either directly stated or implied |
| Community-level assets | |
| Social support | Social support received or provided-but not just access to community support (coded as community connections)  Practical, emotional, spiritual, personal care provision, liaising/organising as forms of social support  Examples of practices carried out by community related to cultural norms, customs, rituals or obligations to community and who is responsible for them, whether some groups are burdened more than others, under what circumstances customs aren’t followed-and impact of this on access to community support  How this enhances well-being either directly stated or implied |
| Social capital | Support, action, resources, information, networking or trust stemming from a collective sense of community, accessible to the community with the potential to benefit all in the community  Community action/agency/empowerment/collective-determination  Examples of capacity building to provide for community or community capacities that have been built collectively  Bridging social capital: links between communities with similar status and power but different identities  Bonded social capital: strong links within communities or networks based on similar identities and characteristics  How this enhances well-being either directly stated or implied for individuals, families or communities |
| Community structures | Community structure supportive of social capital and social support- such as community spaces, places of worship, community organisations, libraries, activities, transnational spaces, burial plots  How this enhances well-being either directly stated or implied |
| Population-level assets | |
| Access to services | Specific service types that enhance wellbeing for individuals, families or communities  How this enhances well-being either directly stated or implied |
| Service approaches | Approaches to providing health and community services, models of service delivery, policies, interventions, practices related to end-of-life care and bereavement  eg. Culturally sensitive service provision, spiritual care, trauma-informed care, hospice care, mechanical ventilation, advanced care planning, use of complementary medicines  Interactions between patients, families and service providers related to the approach used  Approaches where services train volunteers to provide care  How this enhances well-being either directly stated or implied |
| Workforce capacity | Health or other service taff characteristics, attributes and qualifications needed to enhance well-being  Training required  Training of volunteers to provide end of life care in the community  Factors affecting workforce capacity  How this enhances well-being either directly stated or implied |
| Access to information | Information that enhances wellbeing through enabling access to end-of-life care and support or community action  May include information about legal frameworks governing end-of-life care practices, mourning and burial  May include volunteer training to provide end-of-life care in the community |
