# Supplementary material for: Enhancing the wellbeing of refugees living with advanced life-limiting illness in high-income resettlement countries: A systematic review
Source: Palliat Med. 2025 Jun 14;39(7):750–64. doi: 10.1177/02692163251338583 (PMC12227814; doi:10.1177/02692163251338583)
Supplement: sj-docx-3-pmj-10.1177_02692163251338583 – Supplemental material for Enhancing the wellbeing of refugees living with advanced life-limiting illness in high-income resettlement countries: A systematic review [file sj-docx-3-pmj-10.1177_02692163251338583.docx]

Supplementary Table C: CASP Quality appraisal of qualitative and case studies

|  | **Aims** | **Methods** | **Design** | **Recruitment** | **Data collection** | **Researcher relationship** | **Ethics** | **Data analysis** | **Findings** | **Research value** | **Rating** |
| --- | --- | --- | --- | --- | --- | --- | --- | --- | --- | --- | --- |
| Abel 2022^1^ | Yes | Yes | Yes | Yes | Yes | Yes | No | No | Yes | Yes | Fair* |
| Bird 2019^2^ | Yes | Yes | Yes | Yes | Yes | Yes | Yes | Yes | Yes | Yes | Good |
| Borneman 2014^3^ | Yes | Yes | Yes | Yes | Yes | Yes | No | Yes | Yes | Yes | Good* |
| Hiruy & Mwanri 2014^4^ | Yes | Yes | Yes | Yes | Yes | Yes | No | No | Yes | Yes | Fair* |
| Hudson 2019^5, 6^ | Yes | Yes | Yes | Yes | Yes | Yes | No | Yes | Yes | Yes | Good* |
| Kristiansen et al. 2016^7^ | Yes | Yes | Yes | Yes | Yes | Yes | No | Yes | Yes | Yes | Good* |
| Sneesby et al. 2011^8^ | Yes | Yes | Yes | Yes | Yes | Yes | Yes | Yes | Yes | Yes | Good |
| Stahnke & Cooley 2022 ^9^ | Yes | Yes | Yes | Yes | Yes | Yes | Yes | Yes | Yes | Yes | Good* |
| Swetz et al. 2011^10^ | Yes | Yes | Yes | No | No | Yes | No | No | No | Yes | Poor* |

* Case studies: See Supplementary Table D for additional items

Rating Key

Good: >8 Yes; Must have appropriate methods, design, data collection, rigorous data analysis, findings and research value

Fair: 6-7 Yes; Lacking sufficient detail regarding aims, methods, design, data collection, analysis, and/or findings

Poor: <5 Yes; Little or no information about methods, data collection, analysis or findings

1. Abel J. Trains to Life – Trains to Death. *Palliative Care and Social Practice* 2022; 16: 26323524221137603. DOI: 10.1177/26323524221137603.

2. Bird JN. Death and dying in a Karen refugee community: An overlooked challenge in the resettlement process. *Ethnography* 2019; 20: 443-462.

3. Borneman T. Spiritual assessment in a patient with lung cancer. *Journal of the Advanced Practitioner in Oncology* 2014; 5: 448.

4. Hiruy K and Mwanri L. End-of-life experiences and expectations of Africans in Australia: Cultural implications for palliative and hospice care. *Nursing ethics* 2014; 21: 187-197.

5. Hudson S. Bosnian communities: understanding and supporting the refugee experience of end of life care. In: 2019, p.A15. BMJ Supportive and Palliative Care.

6. Hudson S. *Personal communication*. 12/07/2024 2024.

7. Kristiansen M, Younis T, Hassani A, et al. Experiencing loss: A Muslim widow’s bereavement narrative. *Journal of Religion and Health* 2016; 55: 226-240.

8. Sneesby L, Satchell R, Good P, et al. Death and dying in Australia: perceptions of a Sudanese community. *Journal of advanced nursing* 2011; 67: 2696-2702.

9. Stahnke B and Cooley ME. End-of-life case study: The use of narrative therapy on a Holocaust survivor with lifelong depression. *Journal of Contemporary Psychotherapy* 2022; 52: 191-198.

10. Swetz KM, Jama AA, Quirindongo-Cedeno O, et al. Meeting the needs of Somali patients at the end of life. *Minnesota medicine* 2011; 94: 43.
