# Supplementary material for: Enhancing the wellbeing of refugees living with advanced life-limiting illness in high-income resettlement countries: A systematic review
Source: Palliat Med. 2025 Jun 14;39(7):750–64. doi: 10.1177/02692163251338583 (PMC12227814; doi:10.1177/02692163251338583)
Supplement: sj-docx-4-pmj-10.1177_02692163251338583 – Supplemental material for Enhancing the wellbeing of refugees living with advanced life-limiting illness in high-income resettlement countries: A systematic review [file sj-docx-4-pmj-10.1177_02692163251338583.docx]

Supplementary Table D: Qualitative appraisal: Additional items for case studies (Hyett et al. 2014)

|  | Reason given for case selection | Adequate contextual description | Adequate case description | Rating |
| --- | --- | --- | --- | --- |
| Abel 2022 | Yes | Yes | No | Fair |
| Borneman 2014 | Yes | Yes | Yes | Good |
| Hiruy & Mwanri 2014 | Yes | Yes | No | Fair |
| Hudson 2019 | Yes | Yes | Yes | Good |
| Kristiansen et al. 2016 | Yes | Yes | Yes | Good |
| Stahnke & Cooley 2022 | Yes | Yes | Yes | Good |
| Swetz et al. 2011 | Yes | No | No | Poor |

Rating key

| Rating | Criteria |
| --- | --- |
| Good | 3/3 Yes |
| Fair | 2/3 Yes |
| Poor | 1/3 Yes |
